# Comparative Efficacy in Challenge Dose Models of a Toxin Expressing Whole-Cell Vaccine against Eight Serovars of *Actinobacillus pleuropneumoniae* in Pigs

**DOI:** 10.3390/ani12233244

**Published:** 2022-11-23

**Authors:** Preben Mortensen, Nils Toft, István Kiss, Vilmos Palya, Han Smits, Miklós Tenk

**Affiliations:** 1 Ceva Animal Health, Corporate Swine, 33500 Libourne, France; 2 IQinAbox ApS, DK-3500 Værløse, Denmark; 3 Ceva-Phylaxia Co. Ltd., H-1143 Budapest, Hungary; 4 Ceva Animal Health, SID, 33500 Libourne, France; 5 Department of Microbiology and Infectious Diseases, University of Veterinary Medicine, H-1581 Budapest, Hungary

**Keywords:** swine, pleuropneumonia, lung lesion score, vaccine, protection, *Actinobacillus pleuropneumoniae*, serovar independent, *Coglapix*

## Abstract

**Simple Summary:**

Vaccine use is considered an integral control method to prevent respiratory disease in pigs caused by the economically significant *Actinobacillus pleuropneumoniae*. As 19 different distinct subtypes (serovars) of this bacterium exists, and several of these can be present at the same time in the same farm, effective serovar-independent protection is desirable. Vaccines based on the killed bacterial contents of a few of the serovars, including three virulent toxins normally produced during the natural infection of pigs, may potentially provide cross-serovar protection. However, little data is available on multi-serovar vaccine protection. Such a commercially available vaccine, the C-vaccine (Coglapix^®^, Ceva, France) was tested in a total of 13 similar infection studies mimicking on-farm situations with the most common serovars (1, 2, 4, 5, 6, 7, 9/11, and 13) in trials with identical design of detailed lung lesion investigations. Reliability of trial design was tested by high reproducibility in different studies on same serovar. The C-vaccine was producing highly significant protection against lung lesions following all serovar-infections. The trial design was determined highly reliable. We conclude that the C-vaccine gives high serovar-independent protection against disease and is suitable for this use in the field.

**Abstract:**

*Actinobacillus pleuropneumoniae* is a major economically significant bacterial respiratory pig pathogen, and whole cell vaccines are used to prevent disease. However, there is little data available on multi-serovar whole cell vaccine protection. Therefore, we determined the protective efficacies of a whole-cell *A. pleuropneumoniae* serovar 1 and 2 vaccine comprising ApxI-III toxins (C-vaccine, Coglapix^®^, Ceva, France) against serovars 1, 2, 4, 5, 6, 7, 9/11, and 13. The infection doses used induced disease representative of endemic field conditions, and standard protocols were used for all studies. Protection against homologous serovars 1 and 2 significantly reduced lung lesion scores (LLS) compared to positive controls: *p* = 0.00007 and *p* = 0.00124, respectively. The protection against heterologous serovars 4, 5, 6, 7, 9/11, and 13 also significantly reduced LLS: range *p* = 2.9 × 10^−10^ to *p* = 0.00953. As adjudged by the estimated random effect, reproducibility between studies was high. A highly significant serovar-independent reduction of pathological lung lesions by the C-vaccine was found for all the serovars tested (1, 2, 4, 5, 6, 7, 9/11, and 13). We conclude that the C-vaccine gives high serovar-independent protection against disease and is suitable for this use in the field.

## 1. Introduction

*Actinobacillus pleuropneumoniae*, the aetiological agent of swine pleuropneumonia, is responsible for high morbidity and potentially high mortality, causing substantial economic losses in the global pork industry. The peracute and acute forms are easily diagnosed due to the evident and distinct clinical signs and appearance of dead pigs. Also, the chronic form, likely to develop from any form of pleuropneumonia, is easily diagnosed via slaughterhouse investigations [1,2,3,4]. Depending on the quality of the on-farm monitoring of animals, the far less distinct clinical signs and low fatality of the subacute form are easily missed and erroneously considered as subclinical; interpreted as “no pleuropneumonic issues”. Even truly subclinical pleuropneumonia will involve pathological pneumonic lesions [3,5], and despite the lack of clinical signs, average daily weight gain (ADWG) and feed efficiency can be negatively affected [5]. These parameters are often further reduced due to development of chronic lesions, commonly seen at the abattoir [6]. Focusing only on clinical signs in an *A. pleuropneumoniae* endemic farm, where all different manifestations are in principle present in a herd over time, will not reveal the full pleuropneumonic impact [7,8]. Lung lesion scoring is considered highly relevant for estimating severity and losses of respiratory disease, such as caused by *A. pleuropneumoniae*, at the farm level [9,10,11,12]. To investigate pleuropneumonia in all its possible manifestations, pathological evaluation of lung lesions appears to be the least biased method. Performing this evaluation close to pneumonic infection would seem to reveal the most accurate validation of the degree of pleuropneumonic impact on the individual pig and is widely accepted as the endpoint measure of *A. pleuropneumoniae* induced disease [13,14,15,16,17,18,19,20,21,22,23,24,25]. In challenge studies, a dose-response relation is shown [13,17,18,26] and any stage of disease from absolute mortality [14,15,16,17,27,28], even in bacterin vaccinated pigs [16], to subacute [13,19] and subclinical pleuropneumonia [13,16,19]. However, high variation in disease inside challenged groups was evident [13,16,17,18,19,22,23,24,25,26].

In many cases the exact *A. pleuropneumoniae* infection status of the individual pig production unit is unknown to the farmer, however the bacterium is endemic world-wide being present in 80–90% of swine farms, up to seven different serovars having been reported on the same farm [29]. The prevalence of serovars varies between countries, regions of countries, and by year of investigation [29,30,31,32,33]. So far, 19 *A. pleuropneumoniae* serovars have been classified worldwide [34]. In reality there are likely 18 serovars, as serovars 9 and 11 can be considered as one: serovar 9/11, as the difference in the complete capsule polysaccharide loci is only one amino acid and they have identical toxin profiles (ApxI, ApxII) [35]. Different serovars are considered to have quite variable inherited virulence partly due to different Apx-toxin profiles [2,29,30,31,36,37].

*A. pleuropneumoniae* has several virulence factors, some are well described, and several are under investigation. The three exotoxins: ApxI-III and lipopolysaccharide (LPS) are of major importance both in the development of lung lesions and protective immunity [2,37,38]; ApxI, II, and III are, together, the antigens considered capable of inducing cross-protection [1,2,3,37,39]. Many other virulence factors have been described [37,39], including membrane proteins, some of which are immunogenic and therefore can add to the vaccine protective capacity [40]. ApxIV, which is only produced during pneumonic infection, is considered to have a role in both disease and protective immunity [37,41].

Modified live vaccines are currently under investigation on an experimental basis only. Their potential as commercial vaccines need further investigation [2]. Several commercial vaccines are available which differ in their composition and can be classified broadly into one of three categories: (1) killed *A. pleuropneumoniae* whole-cell components only (bacterins, including autogenous vaccines); (2) subunit vaccines containing ApxI-III toxins only; and (3) a combination of these [40]. With distinct differences in efficiency, they all reduce clinical signs, but none can fully prevent infection and colonization [42]. Antibodies against ApxI-III are responsible for the serovar-independent protection against lung lesions [3,6,39,40]. Due to limited cross protection between the serovars, bacterin vaccines lack efficacy compared to ApxI-III combined bacterin vaccines, and pure toxoid vaccines lack general protective capacity due to lack of LPS and other cell wall components [3,38,43,44,45].

A combination of the three exotoxins, ApxI-III with LPS, and likely more of the abundant cell-wall based antigens [3,37,43,45], induce a strong and specific cell mediated immune response that can confer serovar independent protection [3]. This is considered a design for an efficacious serovar-independent vaccine, feasible for *A. pleuropneumoniae* prophylaxis to: increase animal well-being, reduce antimicrobial use, and reduce losses due to pleuropneumonia in all its manifestations at any *A. pleuropneumoniae*-endemic farm at any time [3,6,40]. It is evident that a cross-protective (serovar-independent) *A. pleuropneumoniae* vaccine with high protective capacity is desirable for the global swine production. Nonetheless, whole-cell based vaccines including Apx toxins are questioned on their ability to confer heterologous, *A. pleuropneumonia* cross-protection [40,46,47]. Also, partly for logistic reasons (time, expense, number of animals), there is comparatively little data available on the efficacy of such vaccines against different serovars.

The primary aim of this multi-study analysis was to compare the extent of protection against multiple prominent *A. pleuropneumoniae* serovars provided by a vaccine (the C-vaccine) comprising whole cell components of *A. pleuropneumoniae* serovars 1 and 2 together with ApxI, ApxII and ApxIII expressed during the production process. The aim was achieved by comparison of C-vaccine trials carried out over a 9-year period using a standard protocol based on a predetermined individual challenge dose of eight serovars, measuring the reduction in lung lesions using standardised and repeatable models. The data available also allowed estimation of the relative virulence between serovars.

## 2. Materials and Methods

### 2.1. Ethics Declarations

The trial designs were all the same and in close accordance with the European Pharmacopoeia [48]. All trials were performed by Ceva Research and Development (R & D) Department or Ceva Scientific Support and Investigation Unit (SSIU) in Hungary.

All studies followed local law and regulations. Authorization was provided by the Government Office of Baranya County Food Chain-Safety and Animal Health Department, Hungary. Individual study approval IDs are noted in Table 1.

The studies were conducted in accordance with the provision Directive 2010/63/EU (still in force), Hungarian act XXVII/1998 (still in force, with regular updates in content) and the Hungarian Ministerial Decree No. 243/1998, replaced the 15.04.2013 by the 40/2013. (II. 14.) Hungarian Governmental Decree prepared according to the Directive 2010/63/EU.

### 2.2. Selection of Trials

To be able to demonstrate the widest possible range of *A. pleuropneumoniae* serovar challenge studies and provide the highest possible reliability in study, trials selected for this multi-serovar analysis all had the same challenge trial design in accordance with the European Pharmacopoeia [48] and identical protocols for lung lesion scoring. All trials were performed by Ceva Research and Development (R & D) or Ceva Scientific Support and Investigation Unit (SSIU) in Hungary. A total of 13 studies each including one of the eight *A. pleuropneumoniae* serovars 1, 2, 4, 5, 6, 7, 9/11, and 13 performed over the period of 2011 to 2020 were available (Table 1).

In an attempt to provide the most comprehensive reflection of on-farm *A. pleuropneumoniae* endemic situations, a standardised weighted lung lesion score (LLS) was selected as the endpoint. At a practical level, due to the variety in serovar virulence, the impact of dead pigs on the LLS standard deviation and the number of pigs possible to be included per trial and serovar, trials providing a “medium” impact, i.e., being non-devastating but still causing mortality, were selected.

Where multiple studies with the same serovar were available, the weighted LLSs of the challenged vaccinated pigs (Vac) versus the challenged non-vaccinated pigs in the positive control group (Pos) were pooled and analysed while taking the potential effect of study into account (Table 1). Also, variance between studies on the same serovars were analysed to estimate quality of repeatability of the aerosol chamber (AC) challenge model, hence the reliability of data.

### 2.3. Test Centre and Pig Sources

All studies were performed at a high-biosecurity, professional swine test centre (Prophyl Ltd., Mohács, Hungary). Pigs were recruited from one of two high-health, high-biosecurity farms that monitor clinical signs daily and carry out regular infectious disease testing (see inclusion criteria). On-farm post-mortems are regularly performed on fatalities, and incidents of concern are subject to laboratory investigation. The farms were, and still are, free of *A. pleuropneumoniae*, *Mycoplasma hyopneumoniae*, toxin positive *Pasteurella multocida* (progressive atrophic rhinitis), *Porcine reproductive* and *respiratory syndrome virus*, *Aujeszky’s disease virus*, *Classical swine fever virus* and *African swine fever virus*, based on regular PCR and/or serology tests performed either by government university or private labs. *Actinobacillus suis* has never been diagnosed at the farms either by clinical signs, post-mortem, or culturing. Routine testing for *Swine influenza virus* was not carried out but included as part of the standard diagnostics following clinical signs of respiratory disease. Piglets were not used if they had any respiratory or any other clinical signs.

Pigs at 5–6 weeks of age of either sex and of different breeds (Table 1) from one of the two high-health pig source farms, and that had been declared free of infectious disease (see above) were recruited into the studies. Animals also had to have no previous clinical history of infection by *Streptococcus suis* and *Glaesserella parasuis*.

Any animal selected was serum negative in the ApxIV ELISA (IDEXX APP-ApxIV Ab) test and retested at the end of the trial. ApxIV is an immunogenic protein that is *A. pleuropneumoniae*-specific and produced by all serovars [49]. This, together with history, based on careful disease monitoring on the source farm substantially reduces the risk of non-sero-converters and “hidden” carrier pigs.

### 2.4. The Vaccine

The vaccine tested was Coglapix^®^ (Ceva Santé Animale, Libourne, France), hereafter referred to as C-vaccine, which comprises whole cells of *A. pleuropneumoniae* serovars 1 and 2 expressing ApxI, ApxII and ApxIII, during the production process. Apart from the cross-protective Apx-toxins, this vaccine contains all principal cell wall structures of *A. pleuropneumoniae* in undetermined quantities which contribute to *A. pleuropneumoniae*-protective immune responses: LPS, outer membrane proteins and several other cell wall components; all details available on the EMA web site [50].

Over the span of the 9 years of these studies, the vaccine composition and quality control did not change.

### 2.5. Characterisation and Preparation of the Challenge Strains

The challenge strains were all field strains isolated from pigs that had died in severe acute outbreaks of swine pleuropneumonia, and all were considered as clinically virulent by local stake holders (veterinarians, farm owners/managers and staff) (Table 1). The serovar of all the *A. pleuropneumoniae* challenge strains was confirmed in a multiplex-PCR based on capsular loci carried out as described previously [34,51].

Strains were assessed for their ability of growth in liquid culture in a Tryptic soy broth supplemented with yeast extract and nicotinamide adenine dinucleotide solution in shake-flasks rotated at 180 rpm and kept at 37 °C. Their growth curve was analysed using sampling at pre-determined sampling points and subsequent optical density (OD) measuring at different wavelengths using a standard laboratory photometer. At each sampling point, the cultures were subjected to colony forming unit (CFU) counts using standard bacteriological techniques. The OD and CFU values were then aligned, and the strain-specific, optimal wavelengths were determined. After this initial procedure, in each case when a challenge trial was performed, the strain used was prepared in shake flasks under regular OD monitoring and stopped when reaching the desired live titre based on the OD-CFU calibration curve.

### 2.6. Aerosol Dosing Technique

A standardised AC model developed at Ceva Phylaxia by V. Palya and J. Benyeda, inspired by previous AC work, was used [13,27,52]. The system consists of a box, a nebulizer and tubing with fan to transport the aerosol to the chamber. The plywood box has doors on both sides enabling one way movement of the animals during the challenge. Also, it has an acrylic observation window and a slot for aerosol sample collection. Aerosol is produced by an ULTRAfogger ^TM^ P4 nebulizer (ME International Installations GmbH, Achim, Germany), attached with experimental sample container, tubing and a fan to transport the mist into the aerosol chamber.

Challenge strains were propagated and used for the test when 10^9^ CFU/mL concentration was reached. The *A. pleuropneumoniae* stock was diluted in sterile PBS to achieve the optimal required 10^6^, 10^7^ or 10^8^ CFU/animal treatment dosage as shown in Table 1. Actual calculations were made at the test site, using the following parameters to introduce a definite dose/animal during the aerosol treatment in the chamber:

- Pig body weight [53] and volume;
- Number of pigs placed in the chamber for one run (6–10);
- Volume of chamber;
- Volume of liquid, turned to aerosol by the ultrasonic nebulizer in 10 min (usually 100–150 mL, depending on air temperature and humidity).

Before the first run of a serovar challenge, the AC was moisturized by running the nebulizer with cold distilled water, to avoid precipitation of challenge material onto dry surfaces. Before each run piglets introduced to the AC were given a couple of minutes of ease to ensure normal respiration before the doors were closed and the challenge initiated. The pigs were evenly distributed and secured in the AC by partition fences; the aerosol created by the nebulizer was uniformly dispersed by internal ventilation. After 10 min of treatment, the pigs were kept in the AC for an additional 2 min with the nebulizer switched off, to allow complete uptake of the aerosol droplets (fresh air was provided during this time to allow normal breathing). Between trials the box and the nebulizer were thoroughly rinsed with water, disinfected with Virkon S, water-rinsed again, and left to fully dry out.

### 2.7. Intranasal (IN) Challenge

Production of the challenge strain and calibration of the challenge dose was as described above. The cultures were prepared in 10× concentration of the desired challenge titre and diluted in sterile PBS to reach the working concentration. Each animal received 5 mL of challenge dose into each nostril using intranasal cannulas; the exact individual animal dose is shown in Table 1.

### 2.8. Determination of Individual A. pleuropneumoniae Strain Challenge Dose

Prior to using the strains in vaccine challenge trials, but not as part of these trials, challenge dose calibration studies were performed. In these trials, three groups of 10 non-vaccinated *A. pleuropneumoniae*-negative pigs were challenged with doses of 10^6^, 10^7^ or 10^8^ CFUs, monitored daily for clinical signs and euthanized one week later. Mortality and LLS were evaluated to select the optimal challenge dose. The best challenge dose was the one on being non-devastating but still capable of causing mortality, thereby also reflecting on-farm *A. pleuropneumoniae* endemic situations.

On top of that, for *A. pleuropneumoniae* serovar 2 and *A. pleuropneumoniae* serovar 9/11 the pleuropneumonic impact of different concentrations of challenge dose were investigated via LLS to evaluate the *A. pleuropneumoniae* protective capabilities of the vaccine for different challenge loads.

### 2.9. Trial Design

All challenge trials were performed using the same overall study design; criteria as specified above. The only difference was the use of a standardised AC challenge model by SSIU and IN application by R & D detailed above and in the overview (Table 1).

In all studies, pigs at the age of 6–7 weeks were randomly assigned to either a vaccinated and challenged group (Vac), or a non-vaccinated and challenged, positive control group (Pos). In the AC model studies, a non-vaccinated, non-challenged negative control group (Neg) was included.

Pigs were housed indoors, with controlled temperature and ventilation. Groups were allocated to different pens in the same barn; without direct contact but sharing same air space.

Until 2019, group sizes were chosen only to comply with the requirements of the European pharmacopeia: minimum 7 pigs in each of the Vac and Pos groups; no Neg group required [48]. From 2019, onwards, following advice from a statistician, group sizes of Vac and Pos were increased to 20, to acquire a statistical power of at least 80% based on calculation on the previous trials. The Neg group included in the AC model challenges were chosen as half the size of the Vac and Pos groups.

Each pig of the vaccine group (Vac) received the first 2 mL dose of the C-vaccine by intramuscular injection (D0), at an age of 6–7 weeks. Three weeks later, D21, the pigs of the Vac group received a second 2 mL dose intramuscular of the same vaccine; the Pos and Neg group pigs received no treatment. Pigs were randomised according to bodyweight and staff responsible for the daily care and monitoring of the pigs were not involved in vaccination and unaware of which pigs belonged to which test-groups.

At D42, 12–13 weeks-of-age, all pigs individually received pre-determined equal doses of the relevant virulent *A. pleuropneumoniae* strains either by application in an AC, or by the IN route, as described above.

At D49, one-week post-challenge, the trials were terminated. All live pigs were humanely euthanized and pathoanatomically evaluated to establish the individual lung lobe lesions to calculate the individual LLS. Persons performing the pathoanatomical evaluation were not involved in vaccination and unaware of which pigs belonged to which test-groups.

For *A. pleuropneumoniae* serovar 2, three studies, for *A. pleuropneumoniae* serovars 4, 6 and 9/11, two studies, and for the remaining *A. pleuropneumoniae* serovars, one study were included in the analyses.

### 2.10. Post-Mortem Evaluation of Weighted Lung Lesion Score (LLS) and Other Data

In the vaccination-challenge trials, all animals euthanised on day 7 post-challenge (D49) were subjected to necropsy by a pathologist and investigated for all pneumonic pathological lesions including those that are characteristic of actinobacillosis. Evaluation of the post-mortem lesions in the lungs and on the pleura were performed blind and in accordance with a previously described scoring system [10]. All seven lobes of the lung of each pig in trial were examined and each lobe scored on prevalence of pathological lesions of pneumonia and/or pleuritis (pleuropneumonia). Score valuing was according to the size of the affected area: absence = score 0, 1–20% = score 1, 21–40% = score 2, 41–60% = score 3, 61–80% = score 4, and 81–100% = score 5 [10].

Weighting factors were applied on all individual lung-lobe scorings according to the relative size of each lobe in the lung of each pig: right-cranial = 0.07, right-middle = 0.15, right-caudal = 0.35, accessory-lobe = 0.05, left-cranial = 0.04, left-middle = 0.09, and left caudal = 0.25 [54]. Pigs that had died during the week following challenge and before termination of the trial were given the maximum LLS of 5. This way each pig lung ended up with a total LLS of 0–5, the more lesions the higher the score.

### 2.11. Statistical Analyses

The effect of vaccine on LLS was analysed using linear (mixed) models. Each *A. pleuropneumoniae* serovar was analysed using its own separate model. If more than one study was available for an *A. pleuropneumoniae* serovar, a random effect of study to account for the possible clustering of effects within a study was included. To assess the importance of the between-study variation when more than one study was available for an *A. pleuropneumoniae* serovar, the intraclass correlation coefficient (ICC) was calculated as the proportion of the total variance attributed to the random effect of study (σ^2^_study_), i.e., ICC = σ^2^_study_/(σ^2^_study_+ σ^2^_res_), where the total variance was the sum of the random effect of study and the residual variance (σ^2^_res_).

For the outcome (LLS), a limit of detection (LOD) was defined as half the minimum observed LLS. The LOD was added to all LLS before it was log transformed to improve the underlying assumption about normal distribution of data. All analyses were done in R [55], using the lme4 [56] package for statistical analyses of mixed effects models with the lmerTest [57] package for testing of significant effects.

## 3. Results

### 3.1. Serovar-Independent Protection

The protection of the C-vaccine against the homologous *A. pleuropneumoniae* serovars 1 and 2 strains was demonstrated with highly significant reductions of LLS in the Vac group compared to the Pos group: *p* = 0.00007 and *p* = 0.00124 respectively (Table 2). The protection of the C-vaccine against the heterologous *A. pleuropneumoniae* serovars 4, 5, 6, 7, 9/11, and 13 was demonstrated with equally highly significant reductions of LLS in the Vac group compared to the Pos group: *p* = 2.9 × 10^−10^ to *p* = 0.00953 (Table 2). LLS was absent in all Neg groups except some pleurisy in the 2019 serovar 6 group. This group revealed growth of *Streptococcus* spp. from these unexpected lesions.

### 3.2. High Repeatability and Reliability of AC Challenge Model

In all studies applicable (all AC model studies), the Neg group stayed sero-negative in the *A. pleuropneumoniae* ApxIV ELISA, and in all studies except one, all pigs in the Neg group were without any LLS at the time of autopsy; in the second serovar 6 study from 2019 pleurisy (and polyserositis) was observed in the Neg group together with, what was considered unusually increases of pleurisy in both the Vac and Pos groups. Subsequent extended diagnostic investigations in pigs of all three groups revealed growth of *Streptococcus* spp. from such lesions.

Some variation in mean LLS was observed between *A. pleuropneumoniae* serovars, supporting the decision to analyse each *A. pleuropneumoniae* serovar separately (Table 2). For *A. pleuropneumoniae* serovar 2, the ICC = 0.026, i.e., only 2.6% of the total variation was due to differences between studies. For *A. pleuropneumoniae* 9/11 the ICC = 0.022, i.e., 2.2%, and for *A. pleuropneumoniae* serovar 4 ICC = 0.048, i.e., 4.8%. This suggests a standardized set-up, where the effect of study essentially can be ignored in the analyses. However, for *A. pleuropneumoniae* serovar 6, the ICC = 0.35, i.e., 35%, suggesting that there were marked differences between these two studies.

### 3.3. Other Data

Clinical signs including rectal temperature were observed and recorded, but to differing protocols in different trials, and not considered for efficacy evaluation.

## 4. Discussion

The concept of combining the ApxI, ApxII and ApxIII [3,6,39,40] with cellular components of *A. pleuropneumoniae* [38,44] has been demonstrated to result in a highly effective and highly significant reduction in LLS from homologous as well as heterologous serovars in this study. Reduction of mortality as well as improved productive performance have been demonstrated previously in field studies compared to non-vaccinated [7] as well as pigs given a subunit vaccine [58]. The use of a vaccine with these characteristics will increase animal well-being and reduce both antimicrobial use and economic losses due to pleuropneumonia in *A. pleuropneumoniae* endemic swine farms [3,6,40].

Clinical signs have been recorded in our studies as in almost all similar challenge studies. However, the scoring of clinical signs is commonly performed according to different, non-standardised protocols of high variety, preventing comparison between studies. This was unfortunately also the case for our studies. Rectal temperature is under influence of micro-climatic conditions, individual stress level, and individual pigs may have quite variable individual base line temperature. Furthermore rectal temperature is not unambiguously correlated to behaviour, well-being, and appetite. For that reason rectal temperature data was not considered to provide additional value to post-challenge clinical evaluation and lung lesion scoring, in line with other authors [14,15,16,17,18,19,20,21,24,26,27,28,44].

Weighing of pigs, other than at the time of randomisation, was not considered relevant, as the short observation period prevent an ADWG calculation from being a meaningful parameter.

LLS as endpoint data was considered the most comprehensive, reliable (measurable), and valuable parameter in describing the impact of *A. pleuropneumoniae* induced pleuropneumonic disease mimicking the situation on an endemic farm; studies were selected fitting this purpose. A combination of limitations of group sizes/number of studies, and variation in strain virulence prevented mortality from being a relevant parameter. If mortality had been the endpoint, it would have prevented LLS from being a meaningful parameter due to quite extreme variations (standard deviation). Finally, the true mortality can be biased by the commonly short duration of post-challenge investigation, whether humanely euthanasia is performed or not, and the personal threshold of when to euthanise. In small groups, like in *A. pleuropneumoniae* challenge studies one dead pig more or less, have a great impact on mortality rate and subsequent statistical analysis.

To our knowledge, based on publicly available information, this is the most exhaustive testing on any *A. pleuropneumoniae*-vaccine; experimental or commercial. We have analysed the efficacy of the C-vaccine in protecting against lung lesions from field strains of eight different serovars, all isolated from animals in outbreaks considered clinically virulent to all local stake holders, and of high relevance in the swine production at large, i.e., six heterologous serovars (4, 5, 6, 7, 9/11, and 13) and two homologous (1 and 2) on which the vaccine is based. We found a significant reduction in LLS for the Vac groups compared to the Pos groups. This implies that the vaccine is capable of inducing serovar-independent protection, a valuable characteristic for optimizing the control of *A. pleuropneumoniae*-related pig health problems.

The two R & D studies used IN challenge according to requirements of licensing authorities. These studies were included to expand the range of serovars tested. When considering challenge models, for most investigators, the choice is between IN or AC. IN has an inherited accuracy in applied dose but is labour intensive and comparatively more expensive. In addition, dependent on pig handling and dose application (e.g., sedation/non-sedation), IN is potentially more stressful which can increase respiratory rate, hence respiratory volume, and can affect the planned dose. With AC, skilful pig handling can ensure acceptance of the animals to the chamber and less stress. Our results indicate that reproducible protection studies can be performed with AC when using the described standardised AC model in *A. pleuropneumoniae* challenges and to our knowledge is the only one validated for reproducibility using the intraclass correlation coefficient (ICC). The determined, reproducibility of the challenge studies was high, and the data produced are of high reliability. Hence, accurate individual challenge dose calculations, and lung lesion scoring based on standardized methodology [10], adapted to the biological appearance of the lung [54] is reflected in a standardized, reproducible, weighted LLS from both the IN and the standardised AC model used in this multi-study analysis. Subsequently the majority of studies presented here, all post release studies performed by SSIU, and future studies will use the standardised AC model.

The variations attributable to differences between studies (all AC model challenges) are very low: 2.6%, 2.2% and 4.8% for three *A. pleuropneumoniae* serovar 2, two serovar 9/11 and two serovar 4 challenge studies, respectively. An outlier is the 35% of variation attributable to trial between the two serovar 6 challenges. An explanation could be that excessive pleuritis was generally observed in a larger proportion of the animals in the 2019-study. Bacteriology demonstrated the presence of *Streptococcus* spp. in these samples. Significant improvement in LLS compared to the control group was still observed in this trial alone, and even more so when analysed together with the 2018-study. That infection with other pathogens, e.g., *Bordetella pertussis*, can affect lesion score in *A. pleuropneumoniae* challenged animals has been documented by others [25]. The absent lung lesions in all Neg groups, except the one with pleuritis of likely *Streptococcus* spp. origin combined with sustained negative ApxIV serology, indirectly confirms that the lung lesions in the Vac and Pos groups originate from the specific serovar used for challenge only.

Searching for AC challenge studies with *A. pleuropneumoniae* to compare serovar virulence and AC models, sixteen relevant papers were found [13,14,15,16,17,18,19,20,21,22,23,24,25,26,27,28]. In total, five common and important serovars were investigated: seven on serovar 1 [14,15,16,17,26,27,28], four on serovar 7 [21,23,24,25], two on serovar 2 [18,19], one on serovar 5 only [20], one on serovars 2, 5 and 6 [13], and one on serovars 2 and 9 [22]. Serovars 2, 5 and 6 were considered as being of moderate to high virulence [13], but this was based on small numbers of animals being investigated, and the result with serovar 5 can be considered surprising given that this is normally considered as of high virulence [59]. Serovar 7 was considered as moderately virulent [25]. Based on very high doses in identical trial designs, serovars 1 and 5 appear comparable in virulence measured on mortality only [15,20]. When comparing dosage and outcome empirically across the heterogenous trial designs, serovar 1 stands out as the most virulent closely followed by serovars 5 and 9, placing serovars 2, 6 and 7 as moderate to highly virulent. However, most of these studies, like ours, were not designed to reveal differences in virulence, rather dosing was aimed at obtaining similar disease severity distributions in the positive control groups to enable assessment of vaccine protection. Nonetheless, our data indicates broad agreement with the literature in that serovars 1, 5, 9/11 are the most virulent, serovars 2 and 13 of slightly lesser virulence, and serovars 4, 6 and 7 as moderate-to-highly virulent. It should be noted that the serovar 2 isolate we used was from Europe which expresses ApxII and ApxIII being of higher virulence than serovar 2 isolates from North America which typically only express ApxII [30]. A definitive rating of the virulence of different serovars by AC would require fully standardised extensive head-to-head trials to be carried out in a reproducible challenge model similar to that presented and validated in this publication.

Whatever the *A. pleuropneumoniae* serovar, strains over the years tend to cluster closely with very little genomic variation [60,61]. Therefore the time span of up to 9 years between isolation of strain and challenge, up to 14 years until present date, should be considered of little, if any consequence.

In this study we have used the standardised weighted LLS model to assess vaccine efficacy against multiple serovars of *A. pleuropneumoniae* after AC-challenge in all post-release studies, as well as in the IN-challenge studies of R & D. In the 16 AC-challenge papers discussed above, 28 tests groups can be identified, with five reporting mortality and describing lung lesions in general pathological terms [14,25,26,27], three use in-house models considering other organs than the lungs and the pleura (hart + pericardium) [13,16,17], two calculate percentage of lung tissue affected [14,20]. Only seven score lung lesions with the standard scheme of Hannan and colleagues [10,18,19,21,22,23,24,25]. None of them used a weighted LLS which takes into account the size of the individual lung lobes for optimal comparison between pigs and groups.

Also, the days from challenge to scoring varies substantially between the 28 groups: twelve are in the interval of 15 to 22 days and only focused on chronic lesions [14,16,17,18,21,22,23,24,25,28], three are intermediate from 12–14 days [14,28], nine are focused on acute, subacute and subclinical lesions in the interval of 5–7 days [14,15,18,19,20,22,24,26,27], and one was assessed only at 24 h [14]; in three publications comparisons were made between groups where dead animals were not part of the analyses, are not included [17,22,24]. Finally, numbers are predominantly small with only 5/28 groups using 10 animals or above [16,17,25], another five used 8 pigs [14,18,20,21,23], and the majority using less pigs in a test group [13,14,15,17,18,19,22,24,25,26,27,28]. Thus, the variation in both dosing and assessment methodology severely complicates comparisons with other reported AC-challenge studies. Further comparative studies would best be undertaken in a highly standardised model with reproducible methodology, as reported here. Also, further research in methods to validate *A. pleuropneumoniae* induced pleuropneumonic losses in general is key, but of particular interest for further evaluation of the subclinical/subacute forms [3,13,16,19]. In a world of reducing antimicrobial use, the ability to perform exact cost-benefit analyses on different *A. pleuropneumoniae* control strategies are already of great importance, and involvement of systematic LLS evaluation is likely to be an integral component of such schemes [9].

Limitations of this study include: The lack of standardised, comparable, and objective scoring protocols for clinical signs, the practical and statistical limitations in producing highly reliable data on both LLS and mortality from the same challenge study of most, if not all, *A. pleuropneumoniae* serovars, and the lack of challenge strain re-isolation from pathological lesions. However, the latter is mitigated by the monitoring and biosecurity in the farms delivering the test animals as well as the test facility, the lack of lung lesions in the Neg group animals, and their sustained ApxIV negative serology throughout the studies. Thus the LLS can be considered to originate from the specific field strain investigated. In addition, the combination of both IN and AC challenge studies in the same multi-study analysis, could be considered a limitation. However, both challenge models had high dosing reliability and endpoint measurements were to the same standardised, objective, and comparable scoring protocol.

## 5. Conclusions

The C-vaccine was clearly effective, providing serovar-independent and highly significant reductions of LLS in multiple challenges with different *A. pleuropneumoniae* serovars 1, 2, 4, 5, 6, 7, 9/11, and 13, tested in either IN-challenge or a standardised AC-challenge model. In both models measured on standardised data of high reliability. To our knowledge, this is the largest single published report of efficacy against multiple *A. pleuropneumoniae* serovars using a standard validated reproducible protocol. In addition, to our knowledge, the standardised AC model is the only one validated for reproducibility in *A. pleuropneumoniae* challenge studies, providing definitive pig challenge doses and weighted LLS for accurate biological evaluation of disease and vaccine protection against *A. pleuropneumoniae*.

## Figures and Tables

**Table 1:** List of *A. pleuropneumoniae* challenge trials used in in this analysis. Two studies performed by Ceva R & D are intranasal dosed challenges (IN) and the 11 studies performed by Ceva SSIU are all aerosol chamber dosed challenges (AC).

Serovar	Origin	Strain ID	CFU/Pig	Group	Pigs	Year	Pig Breed	Official Approval ID
1	Denmark	App. St1 ch BS5689	4 × 10^8^ IN	Vac	8 *	2012	Hungaro-Seghers	BA01/2005-1/2010
				Vac	8 **			
				Pos	7			
				Neg	n.a.			
2	Hungary	App. St2 ch 2008/3 + 2	1 × 10^6^ AC	Vac	11	2015	Topigs-Norsvin x PIC	BA01/2005 és 1/2010-1/2012
				Pos	11			
				Neg	5			
			1 × 10^7^ AC	Vac	9	2017	Danbred	BA02/2000-43/2017
				Pos	10			
				Neg	5			
			1 × 10^8^ AC	Vac	10	2014	Hungaro-Seghers	BA01/2005 és 1/2010-1/2012
				Pos	10			
				Neg	10			
4	Spain	App.90993	1 × 10^8^ AC	Vac	10	2018	Danbred	BA02/2000-43/2017
				Pos	9			
				Neg	5			
			1 × 10^8^ AC	Vac	20	2020	Danbred	BA02/2000-43/2017
				Pos	20			
				Neg	10			
5	Italy	App St.5 13ITA	1 × 10^6^ AC	Vac	10	2011	Hungaro-Seghers	BA01/2005-1/2010
				Pos	10			
				Neg	10			
6	Denmark	App. J.no. 101059 + 2SP	1 × 10^8^ AC	Vac	10	2018	Danbred	BA02/2000-43/2017
				Pos	9			
				Neg	5			
			1 × 10^8^ AC	Vac	18	2019	Danbred	BA02/2000-43/2017
				Pos	14			
				Neg	10			
7	Hungary	App. St7 ch CH.G-I/7-7/12	2.8 × 10^8^ IN	Vac	17	2012	Hungaro-Seghers	BA01/2005-1/2010
				Pos	17			
				Neg	n.a.			
9/11	Hungary	App. St.9 ch (B-2011)	1 × 10^6^ AC	Vac	11	2012	Hungaro-Seghers	BA01/2005-1/2010
				Vac	10 ^+^			
				Pos	10			
				Neg	6			
			1 × 10^8^ AC	Vac	10	2014	Hungaro-Seghers	BA01/2005 és 1/2010-1/2012
				Pos	10			
				Neg	10			
13	Spain	App.99865 + 1	1 × 10^7^ AC	Vac	20	2020	Danbred	BA02/2000-43/2017
				Pos	20			
				Neg	10			

* & ** Coglapix^®^ in 50 mL and 500 mL presentations, respectively. ^+^ Stability test one week after 1st use.

**Table 2:** Results presented by sample size, mean weighted Lung Lesion Score (LLS) and standard deviation (SD) for the vaccinated, challenged (Vac) groups and the non-vaccinated, challenged (Pos) groups for *A. pleuropneumoniae* serovars 1, 2, 4, 5, 6, 7, 9/11, and 13. The *p*-value is for the test of a difference between Vac and Pos within each *A. pleuropneumoniae* serovar. Significance is considered when the *p* value < 0.05.

	Vac			Pos			
Serovar	*n*	*LLS*	*SD(LLS)*	*n*	*LLS*	*SD(LLS)*	*p*-Value ^1^
1	16	0.23	0.31	7	1.96	1.14	*0.00007*
2	30	0.75	1.22	31	2.11	2.05	*0.00124*
4	30	0.65	0.41	29	1.46	1.25	*0.00044*
5	10	0.18	0.54	10	1.18	1.61	*0.00953*
6	28	0.71	0.60	23	1.56	1.13	*0.00195*
7	17	0.04	0.10	17	1.17	1.15	2.9 × 10^−10^
9/11	31	2.26	1.89	20	3.84	1.82	*0.00663*
13	20	1.03	1.44	20	2.66	1.93	*0.00319*

^1^ All tests of significance were done on the log(LLS) to improve the underlying assumptions of the analysis.

## Data Availability

The data presented in this study are available from the corresponding author on reasonable request.
